# The RUDY study: using digital technologies to enable a research partnership

**DOI:** 10.1038/ejhg.2017.57

**Published:** 2017-04-26

**Authors:** Harriet J A Teare, Joanna Hogg, Jane Kaye, Raashid Luqmani, Elaine Rush, Alison Turner, Laura Watts, Melanie Williams, M Kassim Javaid

**Affiliations:** 1 HeLEX Centre, Nuffield Department of Population Health, University of Oxford, Oxford, UK; 2 Nuffield Department of Orthopaedics, Rheumatology and Musculoskeletal Sciences, University of Oxford, Oxford, UK; 3 RUDY Study Patient Forum

## Abstract

Patients have extensive experience of their disease that can enhance the design and execution of research leading to significant innovations and efficiencies in the research process. The research community on the whole have been slow to adopt practices that enable patients to become active partners in research. Digital technologies are providing the means to do this more easily and so are increasingly being used to interact with patients and involve them in the design and execution of research. The RUDY (Rare UK Diseases of bone, joints and blood vessels) study’s pioneering approach applies a custom-developed electronic platform where patients can contribute information over time about their disease experience, lifestyle and clinical history. This is combined with a state-of-the-art Dynamic Consent model and a commitment to patient-driven research, to further our understanding of rare diseases. This paper describes the RUDY study and the benefits that have been gained from adopting this partnership approach to research.

## Introduction

Digital technologies are increasingly being used to support all aspects of patient engagement, involvement and participation in biomedical research, at all stages along the research pathway,^1^ and in clinical care.^2^ Examples include the use of social media for advertising including online surveys and recruitment,^3^ dynamic consent,^4, 5^ governance tools,^6^ m-health and mobile sensors^7, 8^ and analysis.^9, 10^ The RUDY (Rare UK Diseases of bone, joints and blood vessels) study is a patient partnership where an online platform is being used to support a clinical network for rare diseases in the UK (www.rudystudy.org). This does not simply involve developing electronic versions of existing paper-based systems, but instead has initiated a new approach to biomedical research. Patient partnership is built into the fabric of the research design, relying on a behaviour change from the participants, researchers and clinicians involved. This paper describes how patients are involved in the RUDY study using digital technologies, outlines how patients have been involved in shaping and designing the platform, and highlights the initial experiences and observations.

### The RUDY study objectives

The aim of the RUDY study was to build a clinical research network that could provide a research platform for patients across the UK with rare diseases of the bone, joints and blood vessels. Rare diseases are under-researched, and approaches to clinical care vary considerably, partially due to the lack of information and understanding about such conditions.^11^ Rare diseases included in the RUDY study are those with incidence rates of less than 1 in 2000, resulting in a relatively small patient population.

Patients in the study will be characterised based on quality of life, pain and clinical events as reported by the participants with validation using clinical records. Participants consent to have their data linked to other studies that may include physical examination, blood, urine, genetics and imaging results. The findings from this process will inform novel biomarkers and therapeutic targets. The primary objective is to determine a detailed description of patients with rare diseases of the bone, joints and blood vessels, and identify unique patient subgroups within each disease cohort for more detailed phenotyping. It will provide the opportunity to determine the personal burden and patient impact of rare musculoskeletal diseases using quality of life, pain and functional outcomes. Given the scarcity of information currently available, this will significantly increase our understanding of the rare diseases included in the study. It will also build a research cohort of musculoskeletal patients that could be approached for future studies.

## Materials and methods

The study gathers information directly from patients, to learn about their experience, clinical events (eg, fractures), quality of life and disease burden, and crucially seeks to recruit as many patients as possible throughout the UK. The study approach has therefore drawn upon three specific elements: (1) involving patients in all aspects of study design and development; (2) developing a website to enable easy participation; (3) developing ethics and governance mechanisms to support patient partnership and enable patients to tailor their involvement.

### A research partnership

The RUDY study is an example of a patient partnership where patients are encouraged to be actively involved at all stages of the project’s development. While researchers at the University of Oxford initiated the project, patient organisations, such as the Brittle Bone Society, the Fibrous Dysplasia Support Society, the X-linked Hypophosphataemia Network and Vasculitis UK, (For a complete list of contributing organisations please refer to the study website www.rudystudy.org) were involved from the earliest stages of the design process. The success of the project relies on effective and ongoing engagement with participants. Patients contribute to all decisions relating to the project, using their expertise and disease insights to ensure that it is in line with their priorities and meets the needs of the community. This was the impetus behind many of the decisions about the design and management of the project; for example the custom-developed electronic interface to enable easy contribution from participants throughout the UK. The research team worked closely with patient organisations to determine the short, medium and long-term goals of the project, methods to recruit participants, and to enlist a Patient Forum that is instrumental in determining the direction of the project.

The Patient Forum consists of 21 patients representing a range of diseases, and including parents of paediatric patients. It regularly meets (virtually, using video-conferencing) to discuss key questions that arise from the project team. Face-to-face meetings are held if any aspects of the project need to be discussed in more detail, such as planning the development of specific website content. For example, a recent workshop brought together different stakeholders, including patients, ethicists, clinical geneticists and researchers, to evaluate the requirements for a tool that will allow participants to map their family tree. The intention is for participants to be able to link their data with members of their family (provided both participants agree) to enable comparative research to be conducted, and to allow for more comprehensive economic analysis of the burden of rare diseases. The interdisciplinary discussion raised questions relating to the intended research and user-experience, and also the ethical issues that may arise. Incorporating this range of viewpoints at an early stage in design, and continuing to work with the different partners throughout development and testing, will hopefully result in a more intuitive and efficient tool for data collection.

### www.rudystudy.org

The network is supported by an electronic interface, limiting the need for participants to travel. If biological samples are needed, or in studies with more invasive procedures, patients can visit their local research centres or GP surgeries to participate.

The RUDY study relies on participants uploading detailed information about their disease history, clinical experiences and various aspects of how their condition affects everyday life. This acknowledges that patients are in the best position to provide insight into the diseases being studied and offer a more complete picture of the wider implications of the rare diseases than clinical records alone would provide. Much of this detail is collected via questionnaires completed every 6 months, such as SF36,^12^ PainDetect,^13^ EQ5D-5L;^14^ these provide an indication of progression, and the influence of specific events on general wellbeing.

**Figure 1:**
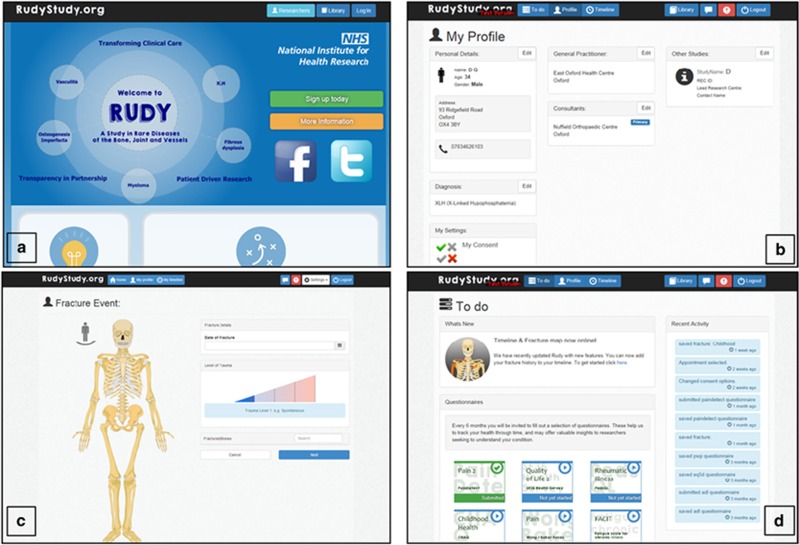
Example pages on the RUDY study platform. (**a**) Rudy front page. (**b**) Participant profile page. (**c**) Fracture event page. (**d**) Participant to do list.

There is also disease-specific content, such as a page for patients with bone diseases to record details of any fractures; for example how the fracture occurred, how they were treated, whether they required surgery, and how well they recovered (Figure 1). Vasculitis patients can record their medication, track changes in prescriptions and dosage, as well as responses to different treatment protocols. The combination of disease-specific information and questionnaires provides a comprehensive record of disease progression, which can be scrutinised by researchers (with approved access) to address specific research questions.

### Ethics and governance mechanisms

**Figure 2:**
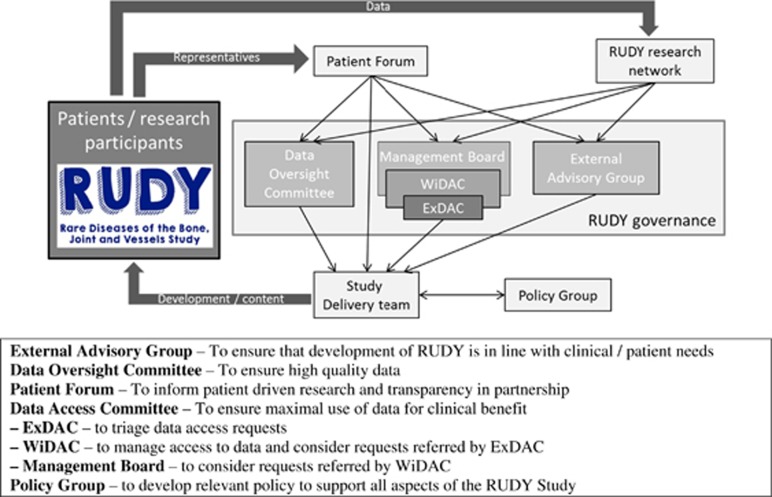
RUDY study governance structure.

The RUDY study is supported by research governance structures that enable clear and transparent oversight of the data, determining how it is used and who has access to what (Figure 2). There is scope for collaboration with industry partners, which will be instrumental in developing new treatments and therapies informed by the data gathered via the RUDY study. How this is managed will be determined on a project basis, in consultation with the Patient Forum, and with specific consent from participants.

The Data Access Committee (DAC)’s aim is to ensure collaborative, maximal use of study information for clinical benefit. Researchers wishing to access RUDY study data submit an application to the DAC (consisting of clinical, technical, ethical and patient representatives, including the principal investigator, and arranged in a tiered committee structure to enable swift review) for consideration against the objectives and ethical approval of the project (and patient consent), the research priorities and other projects that have already received approval for access (encouraging collaboration where appropriate). The data access form is available on the RUDY study Platform in the pages for researchers. The Data Oversight Committee is responsible for ensuring high-quality data, including overseeing changes to the database structure and content, and validation of data entry. The External Advisory Board meets annually to ensure that future development of the database and project is in line with clinical and patient needs. At these meetings the study management group present the activity over the last 12 months and a draft plan for activities in the next 12 months informed by the Patient Forum.

The Patient Forum contributes to all aspects of governance, with representatives central to each of the governance committees that support the study. It provides feedback on specific RUDY methodology questions and tests new website functionality, to ensure that it is useful and working well before being published to the wider study. The benefit of having a specific patient group that provides representatives for the other committees is that it creates a format for patients to discuss key issues, and for the representative to be reassured that they have authority from the Forum to feed into the other committees. Another key tool to support patient empowerment within the project is Dynamic Consent,^4^ which enables participants to review and change consent preferences throughout the project and to tailor their involvement as the project progresses (if for example their circumstances changes). Participants register to take part in the RUDY study via the website (www.rudystudy.org), providing initial information to allow the study team to organise a telephone call. This enables patients to ask questions, and to discuss the consent form, finally giving verbal consent if they are happy to take part. Patients are then sent a copy of the consent form (either by email or through the post) that they sign and return to the project team. The patient information sheets and consent forms are all available online and are emailed in advance, in preparation for the consent conversation. Once the signed consent form has been returned, the project team contacts the patient’s GP or hospital clinician to confirm diagnosis (and thus eligibility for the study) and provides the participant with access to their secure online personal profile. (While most patients participate online, it is possible to receive paper versions of all content) This procedure will soon be updated, having received ethical approval for online consent (discussed further below). The Dynamic Consent record is accessed via the participant’s personal profile, and initially reflects the consent given during the telephone conversation. This record can be viewed and updated at any time, using a simple tick-box system. Any changes to consent preferences are reflected in the permissions associated with the data or samples, instructing the RUDY team on which data items/samples are available for research at the time of data access. Included in the consent preferences are details on how participants would like to be contacted, the frequency with which they receive information and the sort of information they would like.^15^ It also allows participants to determine how their data and samples are used and by whom (including national and international industry partners), and potentially to make decisions about what will happen with their data and samples in the long term, including if they die. The study is divided into sub-studies, which allows participants to select their level of involvement and to have more specific control over how their samples and data are used. Each consent choice has embedded explanations (tooltips), and links to the information library, so that patients can be reminded of exactly what they are agreeing to, or access further information if needed.

Dynamic Consent provides an easy method of reconsent if a relevant new sub-study is added, or a substantial amendment is made to the project. Participants are sent an email with a brief update describing the changes and the new preferences are set to ‘no’ (as a red cross (x)) requiring participants to actively select them or contact the study team to ask further questions. This ensures that patients continue to be in control of their involvement in the study as the research progresses.

## Preliminary results and participants’ experiences

To date the RUDY study has recruited 566 participants (Table 1), with 5461 questionnaires completed (as of 31 January 2017). The information gathered through the online questionnaires has already generated findings relevant for patients with osteogenesis imperfecta, X-linked hypophosphatemia and fibrous dysplasia, as demonstrated by a paper recently published in the Orphanet Journal of Rare Diseases.^16^ The research team has received a number of enquiries from other research groups in the UK and globally; for example we are currently working with colleagues at the University of Osaka Faculty of Medicine in Japan to explore the possibilities for implementing a ‘RUDY Japan’, and with patient representatives from other rare disease groups requesting to be included in the study.

Informal feedback from participants and members of the patient forum suggests that the specific aspects of the project that have been developed to enhance participant experience and engender a partnership between researchers and patients, are working well, and we intend to explore this further with qualitative and quantitative research.

## Discussion

Part of the enthusiasm for the project can be explained by the focus on rare diseases. It is widely recognised that rare disease patients experience significant challenges accessing care; that there is still significant progress to be made in rare disease research; (Rare Disease UK: The Rare Reality—an insight into the patient and family experience of rare disease. www.raredisease.org.uk/media/1588/the-rare-reality-an-insight-into-the-patient-and-family-experience-of-rare-disease.pdf) and that research into rare diseases faces a set of unique challenges presented by the lack of information available and the limited number of patients.^17^ For example, the requirements for specific sample sizes in traditional clinical trial approaches are incredibly difficult to achieve in rare disease populations.^18^ One of the reasons the RUDY study works so well is that this is a uniquely motivated group. Patients experience visits to the hospital where clinicians do not know how best to treat them and may have never previously seen a patient with their diagnosis. There is considerable disagreement between specialists about how best to proceed with standard care and different patients may experience very different manifestations of their disease, with unique sideeffects and challenges. The opportunity to contribute to a project that streamlines information, coordinates efforts and builds a knowledgebase, is compelling.

However, the success of the RUDY study is not just related to the focus of the study (Table 2). Several aspects of the management of the project have contributed to the speed with which the project has progressed and the support that it is receiving. The multidisciplinary research team, which meets weekly to discuss progress and immediate priorities, includes the developers responsible for building the platform. This direct involvement of the information technology specialists enables the platform to be fit-for-purpose, with iterations and new content discussed across the team, including patient representatives. It ensures that the platform meets the needs of both the participants and the researchers, while enabling the developers to manage expectations for what is possible and how long it will take. A balance is struck between which data the researchers are interested in, and what makes sense for the participants. For example, enabling participants to provide a label for fracture events, such as ‘sister’s birthday’ to ensure that they can also use the platform for their own benefit, rather than just setting up a research database to record the ‘fractured tibia’.

The role of the Patient Forum is instrumental in ensuring the success of the study. This constant level of patient input has had a tangible influence on the project, with minimal time burden for the participants involved, with the forum contributing to all decisions, from the study name and logo to the questionnaires included (for example, tracking sleep quality was added following a suggestion from participants), to the priority areas for research. This involvement is vital in ensuring that the website is easy to use, to enable participants to contribute data quickly, and to avoid frustrations if they cannot easily find information or input data (which risks restricting involvement, and limiting the data collected). It also ensures that the RUDY study tackles areas of research that are priorities for the patients that the research is intended to help.

The RUDY study is one of the first examples of a research project that has adopted and implemented a Dynamic Consent approach.^4^ Dynamic Consent enables participants to have greater control over how their samples and data are used in research and enables participants to tailor their levels of involvement and engagement. It is therefore entirely in line with the fundamental tenets of the RUDY study—that the patients are central to the project, and drive the research agenda. The inclusion of Dynamic Consent has been welcomed by participants, and has led to suggested alterations to the project. Early feedback from potential participants voiced a concern about the scale and magnitude of data collected through the RUDY study. Thus Dynamic Consent has been applauded for providing a means to cut the project into manageable pieces, allowing new recruits to get involved in sub-studies, and then gradually expand their involvement if they want to as they become more familiar with the project. This also provides a mechanism for new studies to be added, with participants easily alerted to this addition, or to other amendments that might influence involvement. A recent amendment enabled unaffected family members to be recruited to the project, in order for their data, with the agreement of both parties, to be linked, to provide valuable comparison data. Using a traditional consent process, this could have taken around 6 months to complete, requiring letters to be sent outlining the required changes and asking participants to return their signed forms.^19^ Dynamic Consent allowed for the reconsent process to be completed within a two-week period, as participants were sent an email notification, which directed them to the new section of the Dynamic Consent page on their personal profile. Participants could arrange a phone call if they had any questions about the reconsent options.

Several participants have suggested that Dynamic Consent circumvents the need for a telephone consent process at all, as it clearly explains what is being agreed to, and enables participants to change their mind. Prompted by this feedback, the research team has received ethics approval to set up an electronic consent process, to allow participants to register online, and if (after answering a number of questions about the study) they do not have any concerns or questions about taking part, they can sign up immediately, or opt to have a telephone call with the research team if they prefer. This will have significant implications for the expansion of the RUDY study.

For sub-studies that require participants to attend appointments in person, such as for blood tests and imaging, a face-to-face consent process will remain with opportunity for participants to ask questions or to opt out, without affecting their overall involvement. The use of an electronic interface to support the RUDY study provides specific advantages to the project. Aside from the speed and ease with which participants are able to input data, (which would be particularly arduous, and costly if required to complete and return paper surveys every 6 months, and to complete a diary of disease events on a regular basis), the website enables the research team to share information about the project using a variety of media. The RUDY study team is generating a series of podcasts to explain aspects of the study, including its goals and objectives, and practical information about how participants access their personal profile. They are also exploring the use of other communication tools such as animations, which could be of specific benefit for parents to help explain the study to their children.

The RUDY study provides participants with an electronic personal timeline as a record of all the information that they have contributed to the study. This is accessible on a variety of different devices, including tablet computers and smartphones, allowing participants to access this information at their convenience. Clinicians have reported that patients are already referring to their RUDY timeline during clinic appointments.

The next phase in the RUDY study will be to develop the family map that will enable participants to link with other family members who are also participating in the study; this will include both rare disease patients and non-affected family members. This will provide a vital comparison, particularly for blood relatives, in instances where genetic information can be compared to help elucidate significant variation. Crucially it will also provide the first opportunity where the burden of disease is rigorously tracked across families, providing a comprehensive picture of the true burden of disease, and thus providing vital evidence to the ongoing debate surrounding health economics, and value generated by meeting the immediate costs of care. The Dynamic Consent process will support this data linkage, by providing real-time confirmation of the decisions of both participants involved, to ensure they have consented to the intended use of data. If this relied on a paper-based consent record, it would be logistically very challenging to reassure researchers that all relevant participants supported the intended use of data.

One of the major challenges in rare disease research is the limited number of patients. By developing an electronic platform, the RUDY study has set the foundation for international research collaboration, with the potential for groups in different countries to adopt and translate the software to enable a wider population to be involved. It is hoped that this approach will lead to significant advancement in the understanding and treatment of this group of diseases, and other rare diseases in the future.

### Study limitations

Potential collaborators raise two main concerns when discussing the study, relating to the online interaction with patients and the quality of the data. The electronic interface reduces the need for face-to-face interaction, which is traditionally seen as an important opportunity to allow participants to raise questions. Although patients are provided with clear information about how to contact research team members, it is possible that this invitation will not be taken up, and that questions will not be asked as readily as in face-to-face meetings. At this stage, with the focus on gathering information, it is considered a low-risk project, and face-to-face meetings are needed if samples are collected, or more invasive study elements (eg, radiological procedures) are introduced, enabling participants to raise questions about those, and all aspects of the study. In addition, patients are encouraged to discuss their involvement in the study with their GP, who will have been briefed on the project by a letter from the research team.

The quality of the data is also an area for consideration. The website has been designed to use questionnaires that are patient self-completed and do not need to be administered. In addition, care has been taken to facilitate easy input of accurate data, with relevant data ranges, and reminder windows if certain fields are left empty. Although some patients might provide inaccurate information or have gaps in their record, with the patient’s permission, the research team are also able to access medical records to clarify details. It has been important to strike a balance between the data that researchers will be interested in, and the data that is useful for patients as part of their personal record. Enabling patients to use RUDY as a record of their personal experience will be vital in ensuring that it is maintained. To deliver this, the research team regularly discusses platform development with members of the Patient Forum, to ensure that the data being requested is clear, and that patients are confident with processes for inputting their information, while limiting the opportunity for inaccurate data or for significant variation between how patients report their experiences. The Patient Forum has secure access to a test site for the platform to allow them to test the new features within the platform’s existing features. The long-term success of the project relies on patients contributing data over a long period of time. This also poses a risk to the project. It will be vital to monitor the involvement of patients, to ensure they are completing questionnaires regularly. Keeping patients interested in the study will require regular updates on how it is progressing, including providing access to research papers, and designing new content based on patient input and feedback.

As previously discussed, a major motivation for taking part in the RUDY study is that there is very little research taking place in these disease areas. There is therefore significant need to meet the expectations of participants, and ensure that the data being collected is feeding into relevant research projects. There are over 450 different rare diseases currently eligible for inclusion in the RUDY study, and in some cases patients might enrol in RUDY from disease groups that are not currently being studied. The study team are therefore monitoring the different diagnoses of patients enroled in RUDY, to inform relevant patient organisations if their members are represented. By providing patient organisations with a summary of the data that could be accessed via the RUDY study, it is hoped that this will help these groups to leverage interest from researchers, to promote the need for specific research to be undertaken.

## Conclusions

We present here a novel approach to patient research, linking patient involvement with state-of-the-art ethical frameworks within a patient-facing web-based platform that has been successfully implemented in the UK both in terms of regulatory approval and also recruitment. The full potential of the RUDY study will not be realised until the first treatments and therapies are available to patients, but as the project grows and more participants contribute their data, the research applications will be significant and will address many aspects of lifestyle, burden and care requirements. This could provide invaluable information for the rare diseases, and for wider society, in terms of the links between health and social care, and implications for work, society and the economy.

The next substantial amendment for the study will be to extend the rare diseases included in the study protocol, to enable other patient populations to benefit from this method. Time will show that the success of the project is not just that the RUDY study is filling a vacuum, but that the role of patients in the running of the project and the genuine collaboration between researchers and patients will set up a lasting legacy.

As the project continues, it will be important to formally evaluate the patient experience. Through quantitative and qualitative appraisal, we will gain a better understanding of the elements that are particular to rare diseases, and where other patient groups, or research projects could benefit from the experiences described herein.

The critical mass of data and knowledge collected within the RUDY study will hopefully lead to advances in clinical care, with direct benefits to participants and future generations. It will provide a demonstration of best practice that will be beneficial for other rare disease groups and for chronic and infectious diseases as well. There has already been significant interest in the platform from other groups within the UK and further afield, as it provides an opportunity to engage with a defined patient group and build a research platform in partnership; we invite others to collaborate.

At the same time, the enthusiasm for patients to contribute to the research agenda has become increasingly evident. In 1996 the National Institute for Health Research (NIHR) established an organisation called INVOLVE specifically intended to support and promote patient involvement in NHS, public health and social care research, (For more information on INVOLVE please refer to the website: www.invo.org.uk) and other funding bodies are asking for demonstrations of patient involvement and engagement as part of grant applications. Individuals or patient organisations, in projects often described as ‘citizen science’ or ‘patient-centric initiatives’,^20^ are taking up the research mantle, to address an area of unmet need or fill a gap (Several charities are specifically focused on areas of unmet need, for example Genetic Alliance: www.geneticalliance.org/about. CR-UK contributes significantly to UK research funding: http://scienceblog.cancerresearchuk.org/2011/06/29/near-doubling-of-uk-cancer-research-funding-in-less-than-10-years/. Patientslikeme allows patients to share their experiences: www.patientslikeme.com/about). Web-based information is recognised as a significant tool for individual patient empowerment^21^ and many of the lessons learned for rare diseases would also be relevant for common diseases.

Without being tethered to a recognised research institution it can be difficult for patient organisations to navigate the regulatory and governance hurdles. Some organisations, for example Cancer Research UK (www.cancerresearchuk.org/) and Arthritis Research UK (arthritisresearchuk.org), act as alternative funding bodies, setting the agenda by channelling funding to specific challenges (www.cancerresearchuk.org/funding-for-researchers/research-opportunities-for-cancers-with-substantial-unmet-need). As funders, they are in a position to encourage researchers to include patients in the design of proposals, although it can be difficult to judge when this process is authentic, rather than ‘ticking a box’ to access funds.^22^ In other instances, patients have worked directly with particular researchers with expertise that is of interest and encouraged them to focus on certain questions.^23^ These are perhaps extreme examples of patients helping to drive the research agenda and although willingness to incorporate patient and public involvement and engagement is gathering traction in mainstream biomedical research, it is sometimes unclear how best to go about it and which interventions are most appropriate. More often than not patients are engaged too late in the process, without sufficient funds to support activities that would bring greatest benefit. When a true collaboration is set up between research teams that can channel the benefits of formal research infrastructure and patients whose experiences can shape and drive the research agenda, significant research gains are evident, as the RUDY study shows.

## Figures and Tables

**Table 1:** Breakdown of RUDY participants to date by rare disease type

*Rare disease*	*Number of patients*
Osteogenesis imperfecta (any type)	108
Fibrous dysplasia	80
X-Linked hypophosphataemia	59
Hypophosphatasia	10
Pregnancy-associated osteoporosis	9
Granulomatosis with polyangiitis	51
Eosinophilic granulomatosis with polyangiitis	23
Microscopic polyangiitis	14
Polyarteritis nodosa	3
Takayasu arteritis	7
Other	138
Total	502

**Table 2:** Key features of the RUDY study

*What are the key features of the RUDY study?*
Genuine partnership between patients, clinicians, researchers
Participants, via the Patient Forum, contribute to decisions relating to all aspects of the project
Data contributed and controlled by the participants
Dynamic consent mechanism to enable tailored participation, and to change preferences over time
Sub-study structure to allow selective involvement
Innovative electronic platform design, with tailored content for different disease groups, as determined by the participants
Allow outputs of research to be posted on participants’ secure page
